# Emotion Regulation Focused Family Therapy With Contemporary Families Affected by Information and Communication Technologies

**DOI:** 10.3389/fsoc.2021.633515

**Published:** 2021-03-22

**Authors:** Nathalie Duriez

**Affiliations:** ^1^Laboratory of Psychopathology and Neuropsychology, Paris 8 University, Saint-Denis, France; ^2^Center for Care, Supporta and Prevention in Addictology Monceau, Group SOS, Paris, France

**Keywords:** family relationships, contemporary family, problematic internet use, ambiguous loss, emotion regulation focused family therapy

## Abstract

In the contemporary world, new information and communication technologies (ICTs) have revolutionized family relationships and organization. Mobile phones, tablets, and computers are entrenched in everyday family life. Therefore, families face new challenges with problematic internet use, blurring boundaries between the outside world and the domestic sphere. Sometimes these changes in living together lead to suffering. How do therapists respond to these new challenges faced by contemporary families? Considering the emotion regulation strategies underlying the problematic use of technology, we develop an Emotion Regulation Focused Family Therapy. Within the *Change Process Research* paradigm, which aims to explain how psychotherapy produces change, we examine this innovative therapeutic approach in an exploratory process in order to refine our own practice. We conducted a qualitative research for five families in family therapy under natural clinical conditions to identify the therapist's interventions and the family configurations. The core theme was *therapist interventions*. The results identified 12 subcategories under this category. We built an emotion regulation focused model with 12 steps from these subcategories. Each of the interventions is illustrated with some excerpts from the sessions. Clinical considerations, methodological issues limiting the current body of work, and recommendations for future research are discussed.

## Introduction

Psycho-social mutations have changed family configurations and concerns in family therapy. New ways of “being a family” are emerging: more prevalent stepfamilies, same-sex parenting, late adoption, medically assisted reproduction, etc. Families are also facing changes with the rise in the use of information and communication technologies (ICTs). Smartphone, tablet, computer, connected objects have become part of the family landscape and may be altering aspects of family life, increased even more by the covid-19 pandemic context, with telework and quarantine. Transhumanism asserts the notion that humans can be “augmented” by technology (Leary, 1994; Drexler, 2005; Roux and Coeurnelle, 2016). “The human being can ‘become more human’ thanks to technology: longer life, greater intelligence and creativity.” (Roux and Coeurnelle, 2020). Others denounce the dangers of these cognitive prostheses (Derian, 2013). Augmented Man, “connected self”: information and communication technologies have revolutionized human relations and the organization of families. Can we also talk about “augmented” families? The cell phone has become “an extension of oneself” and has come to interfere in people's daily life, creating a new relational dynamic. What are the challenges connected families are encountering? Although the internet use facilitates our lives, some individuals seem to show problematic Internet use (PIU). Among a variety of detrimental online behaviors, PIU is defined as “excessive or poorly controlled preoccupations, urges or behaviors regarding Internet use that lead to impairment and distress” (Weinstein and Lejoyeux, 2010, p. 277). What are the implications for changing family dynamics, family values, and family processes? In her multitheoretical model describing the process of how technologies are affecting couple and family life, Hertlein (2012) suggests two types of changes in relationships: changes to the family structure of relationships and changes to the family process. If families are changing and technology is part of that change, family clinicians may be challenged by the adoption of emerging technologies. How do family therapists respond to this emerging clinical reality?

From the structural-functional perspective, “family structure is the invisible set of functional demands that organizes the ways in which family members interact” (Minuchin, 1974, p. 52). Family structure is determined by boundaries which are invisible barriers regulating contact between members. When Minuchin (1974) defined boundaries, he was referring to the rules that underpin families' transactional patterns; i.e., how the larger system (the nuclear family) operates, as well as subsystems (parents and children) within it. Subsystems are organized hierarchically: power is distributed appropriately within individuals and between subsystems, making reliance on some members more expected than on others. The parents must assert their authority in a hierarchical structure. The respect of such rules in a family makes belonging possible. Technology use may modify these rules and blur work/family boundaries with negative consequences (Chesley, 2005). For example, when a parent works from home, the rules specific to their work system may take over family rules. The parent, who is usually attentive to their child, may become upset because they are disturbed by their 6-year-old son in their work. As for the child, the rules of online gambling may take precedence over the rules of the home. The adolescent, who is supposed to join their parents for lunch, refuses to leave their room because they are playing with friends online. These bubbles allowed by technology appear as subsystems with sometimes rigid boundaries within the family system, which are governed by different rules.

“Personal communication devices now transform the individual into a communicating cell that inhabits several spaces at the same time, that of the immediate environment and all possible spaces virtually accessible through its technological connections (Guillaume, 1994)” (Caron and Caronia, 2005, p. 255). Different affiliations enter into rivalry. The notion of belonging then appears to be obsolete, individuals being linked less by a common belonging than by connections (Gaillard, 2009). With this possibility of external connections, the modeling of the family as a system seems to be called into question, insofar as family members are no longer bound together by ties that would be hierarchically superior to any other ties (Gaillard et al., 2011). Submission to paternal authority is no longer relevant, young people are their own judge and have authority over themselves. Parents used to be able to control the games they were offering their children. Today children can “download their own games without parental knowledge. This may flip the structure in the family to unfavorably honor the youth or place them in a position of higher power, as youth are frequently more knowledgeable about technology than their parents” (Curtis et al., 2017, p. 114). “The family, the first cell of belonging, is subject to permanent reconfigurations and is going through a crisis of educational transmission. We are thus witnessing a generalized mutation of institutions and social and community rituals, which nourished the social bond by giving meaning and regulating individual behavior” (Lascaux and Couteron, 2015, pp. 165–166). Technologies amplify the phenomenon of individualization of children, who spend less time with their parents. Family cohesion^1^ suffers as a result (Caron and Caronia, 2005). Escots (2011) speaks of an epistemological crisis linked to the change in the ontological status of families.

In new families, the children no longer build their identity based on belonging, with an injunction to heteronomy, but by responding to an injunction to be autonomous: “You alone can and must forge your identity!” Hence a high degree of self-esteem is needed. As beings are structured on an “individual” mode, the belonging bound is almost totally dissolved into a connective one. This connective mode of binding must leave each one with an intact space of personal development. His space having become vital insofar as they impose themselves as supports of the necessary personal visibility, of the sufficient deployment of the self of the individuals, of the presentation of oneself, would say Fourez (2004). Young people no longer construct their identity on the basis of belonging but on individuality, which can rely on digital supports that we can refer to the “technologies of the self,” described by Foucault (1982). Young people tell their stories through social networks, recording their moods and the activities that have punctuated their day. Digital tools make it possible to produce a life story (Amri and Vacaflor, 2010), but the receptors of these messages, traces of the young person's narrative identity, are multiple and uncertain. Computer-mediated social ties, made possible by digital technology, are “floating” (Perriault, 2009) and the identity construction induced by this socio-technical logic will be unstable (Coutant, 2011). New fragilities emerge with these recent processes of individualization: narcissistic disorders, emotional dysregulation, new addictions, etc. (Coutant, 2011; Duriez, 2018a).

The process of deinstitutionalization of the couple and of the family induces “subjectivation” and “affectivation” of the bond. The relationship to the other is very concrete, subjectivated, and “affective,” implying a relationship based on esteem -constantly measured- of the other and of oneself” (Gaillard et al., 2011). Regulations within the family are “no longer in an institutional mode, they are essentially in an emotional mode, so that there is a negotiation process that is as continuous as it is tense” (Gaillard, 2009, p. 18). This change in regulation modes can be a source of suffering for some family members who do not adapt to this connective attitude (Duriez, 2019).

Technology carries changes to the family process, from belonging mode relationships to connective mode relationships. From the interaction-constructionist perspective, family process observes how family members develop relationships and interact with one another through communication, behavior, gestures, rituals (Berger and Kellner, 1970), and emotion regulation strategies (Duriez, 2017). Apart from symptomatic situations (addiction, cyberbullying, minor's access to inappropriate content, etc.), the main problem seems to be time spent using ICT with its consequences on family relationships (Hugues and Hans, 2001; Bacigalupe et al., 2014). With regard to parent-child relationships, for the adolescent, “too much isolation in the virtual world can mean a diminished attachment to parents and family. A too strong link to a machine to the detriment of other relationships with surrounding people, a too long period of time spent in its presence, substitutes, to a certain extent, for certain relationships that are indispensable in the context of social and emotional development” (Derian, 2013, p. 356). The widespread presence of new media technologies in homes may have relational impacts on the couples. Hertlein (2012) examines the consequences for couples and points out the effects of technology in daily family life on intimacy, relationship initiation and formation processes, and relationship maintenance. Chesley (2005) demonstrates that cell phone use over time is linked to increased psychological distress and lower family satisfaction.

For example, during a marital therapy speech, a woman explains that every evening, when the couple could meet once the children went to bed, her husband takes his cell phone to the toilet and disappears for 30 min: “he is there and he is not there.” She then experiences a momentary loss. Although this experience can emerge in the context of divorce, it can also exist when all family members are together at home. The oscillations between presence and absence destabilize the entourage, which must learn to cope with the stress due to the fact that we never know if the other person is actually present and available or if they are locked in their technological bubble and inaccessible (Duriez, 2013). This experience of loss becomes an organizing core of the family dynamic as new hidden games regulate the relationships: one is psychically absent by locking themselves up with the computer, while the other feels the absence, the lack. This experience brings us back to the concept of ambiguous loss described by Boss (1999). In the context of an ambiguous loss, it is very difficult to integrate the other's absence because the other is present. The person is no longer present psychically but they are physically and can also return psychically. The process of “progressive detachment,” the stage of grief for a real loss, is not possible. Connected families must learn to regulate the emotions related to the ambiguity of presence, to the uncertainty of the availability of the other.

How do therapists respond to these new challenges faced by contemporary families? Mesch (2006) proposes a compositional approach which includes work on internet use, self-esteem, family time, and family conflicts to strengthen family cohesiveness. Bacigalupe et al. (2014) propose a strength-based approach to support family relationships. “The confluence of an ambient intelligence, an environment of advanced technology capable of responding intelligently to human needs (Riva, 2005) a connected presence (Licoppe, 2004) and the continuous awareness of others through wireless tools, is fostering a new way for families to relate” (Bacigalupe et al., 2014). He recommends that therapists familiarize themselves with new ICTs. Jordan (2014) proposes to incorporate video games like board games in therapy. Curtis et al. (2017) proposes to accommodate and to highlight the benefits of video games in order to assist the youth in staying engaged and active in the process. They invite parents to do the same. “The caregiver, while being coached by the therapist, should facilitate a conversation with the child about what games they like to play or are interested in” Curtis et al.(2017, p. 115). Liu et al. (2015) explore both the effectiveness of a multi-family group therapy on Internet addiction and the underlying mechanisms of the effectiveness. They identify two mechanisms: (1) improving parent–adolescent communication and closeness, (2) fulfilling adolescents' psychological needs through strengthening their communication and relationship with their parents.

Working on communication difficulties is at the heart of therapeutic approaches, whether it be parent-child communication (Young Park et al., 2014) or communication within the couple (Hertlein, 2012). There may however be a paucity in considering the emotional regulation strategies underlying this miscommunication in family therapy with connected families. Researchers are increasingly studying the correlation of Problematic Internet Use (PIU) with emotional regulation (Zhou et al., 2011; Hormes et al., 2014; Casale et al., 2016; Spada and Marino, 2017; Evren et al., 2018; Wei et al., 2018; Amendola et al., 2019; Hernandez et al., 2019; Karaer and Devrim, 2019; Faghani et al., 2020). Sideli et al. (2017) demonstrate that low self-esteem, high aggression-hostility, and high sociability are significant risk factors for PIU. The Internet allows escape, distraction, extrication from a relationship (Lee, 2014) or procrastination (Hernandez et al., 2019), in short “a form of escapism to avoid certain aspects of their reality” (Hertlein and Hawkins, 2012). Schimmenti and Caretti (2017) show that PIU may be considered as a dissociative experience. They propose the clinical construct of Video-terminal Dissociative Trance (VDT) and discuss its potential usefulness for the assessment and treatment of people who display PIU. “As a defense mechanism, dissociation allows an individual to avoid emotional distress temporarily by screening out excessive or overwhelming stimuli (Bromberg, 1996)” (Schimmenti and Caretti, 2017, p. 65). In our practice with connected families these studies invite us to explore the function of problematic internet use in relation to emotional regulation. How does this use maintain the homeostasis of the family system?

## Materials and Methods

Our research questions are as-follow: What are the impacts of ICTs on family relationships? What are the difficulties encountered by families in relation to internet use? What are the relevant lines of work with living digitally families? How does the therapist act?

Within the new outcome research paradigm focused on identifying the processes of change in patients and on analyzing the actions of the therapist bringing about these changes (Goldfried and Wolfe, 1996), a qualitative and exploratory research approach was chosen. For some researchers, “it is no longer possible to be satisfied with studies that focus on comparing symptoms before and after treatment or not. It is necessary to open the ‘black box’ that worked in between” (Thurin, 2017, p. 57). This is what Greenberg (1986) proposes with the concept of “Change Process Research.” Based on circumstantial study of therapies conducted in “natural” conditions, its perspectives are clinical, practical, and theoretical. At this stage of our research, the objective is not the evaluation of psychotherapy as it is not yet formalized. The objective is to identify the experience of families with ICTs through their discourse, what the family requests and how the therapist responds to it in order to build an emotion regulation-focused model for these wired families. Our research isn't an action research either because the data from completed therapies pre-existed the research.

Our research on change processes (Duriez, 2007) and our clinical practice gave us the opportunity to observe that in certain contexts, even if adapted to the individual, the emotional regulation strategies of some family members can be a source of suffering for other family members to the extent that they might question their own strategies. During the therapy we investigate emotion regulation strategies and how the others react to them. If there is an issue, then our therapeutic intent is to bring family members to a greater acceptance of each other's emotion regulating style, without feeling misunderstood or threatened by the differences in emotion regulation strategies (Duriez, 2017). The hypothesizing process stands out within the therapeutic frame in the context of the intersubjective encounter between the family and the therapist, with the therapist also experiencing her own emotional regulation strategies. We apply the principle of circularity between the therapist and the family, which can be defined as “the capacity of the therapist to conduct his investigation on the basis of feedback from the family in response to the information he solicits about relationships and, therefore, about difference and change” (Selvini et al., 1980, p. 6).

### Setting

The setting for our practicum was a Center for Care, Support and Prevention in Addictology (CCSPA) in Paris which provides a no-fee service to people living in Ile-de-France. Professional referrals to the center are not required; individuals can refer themselves. The CCSPA is operated by the Groupe SOS and serves as a family therapy training facility.

### Families

From the libraries of therapy sessions held by the author, five families have been selected because they mentioned a problematic use of ICTs. Forty-seven video-recorded family therapy sessions were sampled. To protect their identities and to ensure confidentiality, the families were assigned pseudonyms. These five families are presented in Table 1.

**Table 1:** Population.

**Family configuration**	**Number of sessions**	**Presenting issue**
**I Family, Stepfamily** Mr Philippe I, 54 years old Ms Armelle I, 53 years old Carmen, 16 years old Axel, 14 years old Mr I's son from a previous marriage Diego, 25 years old	22	(1) Carmen's smartphone use (2) Axel's video games use (3) Ms. I's cyber infidelities.
**R Family, Stepfamily** Mr Jérémie R, 40 years old Ms Valérie R, 45 years old Lucas, 13 years old Ms R's sons from a previous marriage: Baptiste, 21 years old Simon, 18 years old	2	Video game use for the father and the boys.
**S Family** Mr Olivier S, 55 years old Ms Carla S, 48 years old Opale, 16 years old Logan, 12 years old	15	(1) Opale's smartphone use (2) Logan's video game use
**T Family** Mr Antoine T, 32 years old Ms Amandine T, 25 years old Soukaina, 3 years old	2	Online gambling for Mr. T
**V Family, Divorced family** Ms Catherine V, 56 years old Gauthier, 26 years old Florent, 21 years old	8	Video game use for Gauthier and Florent.

### Researcher

The researcher was the therapist for the families, the author. The double role of being a researcher and a clinician presents advantages, facilitating both the development of clinically relevant research and the dissemination of evidence-based treatments into routine clinical services (Yanos and Ziedonis, 2006). It also presents challenges, with ethical conflicts between the clinical mandate and the methodological demands. To address this issue, therapy and research did not occur at the same moment. During the therapy, the analysis of the sessions was carried out by different groups of psychologists in family therapy training, guided by the clinical mandate. The presence of a recording device and of a video recording the interactions were integral to the family therapy setting. Once completed therapies, research was carried out, guided by the scientific mandate.

### Data Analysis

Change process research (Greenberg, 1986; Watson and McMullen, 2016) includes qualitative methodologies that aim to find meaning in and conceptualize data from the complex interactions of psychotherapeutic work, and subsequently, contribute to theory development. We chose a conventional content analysis (Hsie and Shannon, 2015) generally used with a study design, whose aim is to describe a phenomenon, in this case the therapist's interventions that address the challenges faced by families, who are now using new technologies in an increased way.

Data have been obtained from completed family therapies. But a first data analysis started during the therapy. We work with families with a constant concern to question our practice, according to an Evidence-Based Practice (EBP) stance, which gives opportunities to bridge clinical research and practice, enhancing the knowledge base and improving patient care. We have viewed and reviewed the sessions with different groups of psychologists, who are in family therapy training and also with master students in order to allow the emergence of new insights and to gain a general sense of the therapy process. From this exposure and the case notes, which followed each session, we identify relevant moments related to a problematic use of technology. We visualized the significant sessions for the five families and transcribed the relevant passages. Two master students helped transcribing the sessions for families I and S. We started the initial open coding on paper copies of the transcripts, with categories being noted in the margins. Analytic procedures of content analysis are close to the “line by line” practice of supervision, which allows therapists to reflect on the meanings of clinical material. Then we began *axial coding* to relate categories to their subcategories.

We attempted to assume the credibility of our qualitative findings in a number of ways, including our use of triangulation with our colleagues in the group of family therapy training, with participants during workshops (Duriez, 2018a, 2019) and with master students, using more than one source to analyze the data (Merriam, 1998).

## Results

Two core themes were generated: *the experience of the family with technology* and *therapy techniques*. *Therapy techniques* refer to the exact means therapists used to achieve different therapy intentions. The family's experience is expressed through the therapist's exploratory interventions. Therefore, we have considered a single core theme, *therapist interventions*, that includes both the therapist's investigations to identify the family's experience and therapeutic techniques. Results identified 12 subcategories under the category of *therapist interventions*. Table 2 shows these 12 therapist's interventions. We present these subcategories in the chronological order observed during therapy. For each category, we have distinguished these interventions based on the distinctions made by Tomm (1988) according to the therapist's assumptions (lineal or circular) and the therapist's intents (orienting or influencing).

✓ Lineal questions: The intent of this type of questions is to extend the therapist's understanding. The questions are based on lineal assumptions about the nature of the phenomena.
✓ Circular questions: The intent is also exploratory, but the questions are based on circular assumptions about the nature of the phenomena.
✓ Strategic interventions: The therapist reframes what was said from a circular perspective in order to influence the family in understanding the possible connection between observed behaviors. He expects the family members to take responsibility for the effects of one's actions on the others. The intent behind these questions is predominantly corrective.
✓ Reflexive questions: The therapist invites family members to reflect upon the implications of their current perceptions and actions and to consider new options. She triggers reflexive activity in the family's preexisting belief systems. The intent behind these questions is predominantly facilitative.

**Table 2:** Results.

**Therapist interventions**	**Subcategories**
Exploration of the problem (Lineal questions)	Time-consuming activity Reduction in family time Impact on quality of family time Impact on studies and homework Impact on couple intimacy Impact on the family budget Permeability of the boundary between work and family
Exploration of the complaints (Circular questions)	Feeling of abandonment Lack of support Fear
Exploration of the perspective of the designated patient (Lineal questions)	Pleasure Relaxation Refocusing on oneself Connecting with friends Building a protective bubble Psychic unavailability of the parents Conflicting family environment
The negative interactional cycle (Strategic intervention)	Avoidance strategy amplified by the other person's confrontational strategy Low self-esteem amplified by the anger and disqualifications of the other person
The experience of ambiguous loss (Reflexive questions)	Loss of rituals Relationship shaped by loss Emotional unavailability Rigid boundary Bubble Threat of breakup Evocation of death
Making connections between past and present(Reflexive questions)	Non-elaboration of a previous loss Feeling of repetition Consistency with the past Paradox between the official program and the construction of the world
Reframing the use of technology as an emotional regulation strategy (Reflexive questions)	Time to relax Avoidant coping Behavioral disengagement Extrication from the couple's relationship Escape from the real world Distraction Procrastination Regulate neuroticism Emotion sharing Social support seeking
Building the emotion regulation strategies cycle (Strategic interventions)	Time to relax = > Reduction in family time Distraction = > Impact on homework Avoidant coping = > Lack of support = > Impact on problem solving Behavioral disengagement = > Unsatisfactory intrafamilial communication Extrication from the couple's relationship = > Impact on couple intimacy Escape from the real world = > Non-elaboration of past suffering Procrastination = > Fear for children future Regulate neuroticism = > Reduction in family time Emotion sharing (with pairs) = > Feeling of abandonment (for the mother) Social support seeking (outside) = > Reduction in family time
Opening up earlier trauma wounds (Strategic interventions)	Childhood marked by loss (child abuse, parental separation, etc.) Migration Death
Building a sense of internal security/or autonomy (Strategic interventions)	Better knowledge of each other Exploration of “schema-of-being-with” Respect for intrapersonal differences Adjustment to intrapersonal differences Sharing emotions A new understanding at previous wounds
Implementation of new behaviors (Strategic interventions)	Learning to decompress in another way Building new family rituals Building new narratives New way of communicating Sharing emotions Using technology to serve the family cohesiveness
Changes (Reflexive questions)	Acceptation of emotions Refusal of the legacy of ancestor traumas Awareness Agency Discovery of new emotional regulation strategies Emergence of a stronger self

For each subcategory, we take a few verbatims or illustrative exchanges. We selected certain moments in the therapy according to several criteria: repetition of the evoked theme, the expressed emotion, the quality of reflexivity, and the family feedback about meaningful moments. These exchanges were translated from French for this report by the author, preserving the original phrasing where the sense was clear.

### Exploring the Problem, the Interactional Dynamics of the Family, and the Family Structure

During the first session, the therapist asks everyone for a description of the problem and tracks the sequences of behaviors that they use to explain it. They identify the place of screens in family life and their impact on family time, studies, homework, and couple intimacy. They investigate how ICTs influence the way the family establishes rules, roles, and boundaries. With this information the therapist can map the family's underlying structure. The therapist also assesses the quality of the boundary with the outside world and the permeability of this boundary.

The S family is a wired family. Mr. S., the father, spends a lot of time on the computer. Opale, the 16 years old daughter, spends a lot of time on her smartphone. Logan, the 12 years old son, spends a lot of time playing video games. The father and the children are locked in a technological bubble and the mother suffers from this lack of exchange. She feels abandoned or neglected. The initial request for therapy concerns Opale's excessive use of her cell phone. Opale explains: “Actually when I'm with my friends, my phone doesn't exist. It's just that when I get home, I start using it to chat with my friends.” Her mother complains about the lack of communication with her daughter. Opale describes the evening ritual when she comes home from school: “I say ‘hello’ to her, I come over to give her a kiss, I go to the kitchen, I eat and lock myself in my bedroom and she doesn't see me again until dinner and it actually annoys her because she says ‘you pretend I’m not here!.”' Communication with parents becomes a source of stress. If the boundary separating the whole family from other systems is diffuse at home because Opale maintains a virtual contact with her friends, this boundary becomes rigid when Opale is out in her group of friends because she usually cuts off contact with her parents at these times.

Mr. S works a lot and when he is at home, he works too: “We don't have much time together, me and my wife, me first because I get eaten up by my work, everything administrative, accounting, etc.” “Escape with work limits intimacy within the couple. From time to time on Saturday night, they plan to watch a movie together with the video projector. The children are in their room, playing video games or watching a series. Mr. and Ms. S are each in front of their computer, waiting for the other to finish what they are doing to start the DVD. But they don't tell each other and get caught up in answering e-mails, reading the news, checking social networks and then they realize it's too late to start watching a movie. That's the way it is for all the common activities. Mr. S is constantly connected. Ms. S is waiting for him and ends up going online as well and they don't do anything together anymore. The father is disengaged from his children. The mother is enmeshed with her children. She's over-functioning in area of parenting and household chores.

### Exploring the Complaints

First, the therapist questions the person who initiated the demand for therapy, about their own feelings. Generally, it is the mother who demands therapy and expresses her suffering. She usually complains about the poor quality of communication, the perceived lack of support for educational or household tasks. She expresses a feeling of being disrespected and neglected, and a fear for the future.

Ms. R: I have an expectation because I am experiencing a problem of communication with those around me.
Ms. V: I was the one who initiated the demand. The motive at the beginning, I thought my sons were too addicted to video games and the internet and so on, and that's why we came. (.) Because I felt that the house was a bit like a hotel, that we didn't communicate, we didn't talk about real things, and everyone was a bit in their bubble, including me because I was very busy with my work. It made me unhappy to see that we didn't have deep exchanges with my sons.
Ms. S expresses anger because she doesn't feel supported by her husband in the exercise of parenting. She has to over-adapt. This over-adaptation can go as far as a burnout. Ms. S is concerned about the time her son spends gambling to the detriment of his school obligations. She keeps an eye on him every day to limit the time he spends gambling and regrets not being supported by her husband: “It's only when there's a big problem, something that directly disturbs him, that's when he breaks down, that's when he explodes. But day after day, I'm the one who's behind the children. If I wasn't there, that's it! They would be left to their own devices!.”
Mr. T plays online poker when he's not feeling well. Ms. T is worried about the future:
Ms. T: The fear comes because I have to raise a family with Antoine, I can't accept knowing that if one day he breaks down, he could squander the money on our children's education. The pain, the sadness, the disappointment will be intense. My children are everything to me. Telling me that I won't be able to pay for school because my husband screwed up. that's not even an option!

Sometimes the problem is no longer internet use but the parental response to the problem. Mr. I also had moments of anger toward his children, Carmen and Axel, who are playing game online or chatting with friends rather than doing their homework, which Ms. I blamed on him. He then said one day: “I don't want to have this kind of conflictual relationship with my children anymore, I don't want to take care of their education anymore!” Ms. I felt abandoned in this task: “I'm all alone in being the ‘bad lady,’ at least in setting limits.” Mr. I's anger when there are problems with the children has not disappeared: “When Victor is angry about something, he tells me but not them. I tell him: ‘Go and speak to the right person!.”’

### Asking for the Perspective of the Designated Patient

According to the technique of circularization (Tomm, 1987), the therapist questions the persons designated as carriers of the problem about their feelings. The use of technology may relate to different psychopathological meanings according to the underlying psychological motives: pleasure, relaxation, refocusing on oneself, connecting with friends, building a protective bubble, coping with parents' lack of emotional availability, coping with conflicting family environment.

Besides family communication, several personality traits tend to affect technology use. When someone presents low self-esteem, neuroticism or sensation seeking, the problematic technology use can be increased by the family transactional patterns.

Mr. T: It's a way out at a time t. It's as if I'm making my own parenthesis. I had a kind of weird childhood. I've always had a solitary aspect and I need “my moments.” I was sad, I always put myself in this bubble. It's a habit that I picked up. It's almost intrinsic to my personality.

Gauthier explains that with his brother, they locked themselves in video games to escape conflicts with their mother. If they communicated with their mother, the atmosphere was systematically conflictual. He told to his mother:

GAUTHIER: Being able to count on you was not possible because it was either “get by” or “yes” and that never happened. It doesn't help communication. We got into a system where everyone was in their own bubble and we didn't want to get out of it. For Florent and I, as we are solitary characters, it was comfortable, more comfortable than being exposed to the conflictual environment. We put up walls around us and there was no family life. Mom suffered more from that than we did because we are of a solitary nature, so we could survive. We endured it. (.) In terms of family life, this functioning is deleterious!
Ms. V admits to having had a role in this family functioning: “I think it was good for the three of us because I also have a side like that, being in my bubble.”

### Identifying the Negative Interactional Cycle With the Family

The therapist proposes a circular view of the situation with the intent of getting everyone to assume their responsibility for what is happening. On the one hand the pathological ITCs use provides a refuge from the stress of everyday life for the addicted person but it also prevents the other family members from facing problems together. The therapist explains how the two behaviors, for example escaping work for the husband and aggressive communication for the wife, likely perpetuate one another, or are at least a small part of a larger cycle of behaviors. The therapist identifies the positive feedback loops responsible for maintaining the problem and emotional insecurity. A negative cycle is defined as a predictable interactional pattern that gets repeated and organizes the family around insecurity, rather than vulnerability. Negative cycles are fatiguing and destructive for family functioning. One operates with an avoidance strategy that clashes with the parent; the spouse who operates with a confrontational strategy and as a consequence one or the other is left alone to deal with daily life. Mr. S, trapped in his work addiction, is not receptive to the emotional needs of his wife and his children. Ms. S's anger prevents her from being receptive to the needs of her children. No one responds to the attachment needs of the other. Ms. I continues to fantasize a united family:

Ms. I: When I'm at work, I fantasize about my family. I think when I get home: I'll make dinner, we'll have dinner together, it'll be fun. I come home and then I suddenly feel like everything is flammable. It's true, it's adolescence. In 5 min, it's apocalyptic, everybody's yelling at each other, I find myself with my fork in the air, I find it exhausting. We can no longer talk to each other!

Systemic reframing fosters empathy among family members, facilitates responsiveness, and helps the family deescalate. Defenses fall down, anger and disqualification give way to emotions that are harder to express: fear, hurt, and sadness. The feeling of loss induced by technological bubbles is expressed. Ms. R expresses the feeling of having lost her son to video games.

Ms. R: When Baptiste started playing video games, I said to myself: “That's it! I've lost my son!.” The relationship we had died. Because we had a rather fusional relationship and so I lived our relationship. yes like a separation, a mourning, a small death, a loss in fact. a real loss!

### Exploring the Experience of Ambiguous Loss

The therapist bounces off this experience of loss. Her intent is to help the patient identify their emotions and the consequences of these feelings on their behavior toward others. The therapist explores how the relationship to the screens of some people induces a feeling of ambiguous loss for the other members of the family who suffer from this emotional unavailability. Relationships are shaped by loss. Ms. T says about her husband: “It's as if he wasn't there! I am 8 months pregnant, I could give birth any moment but he doesn't help me shower our daughter.” Her husband, absorbed in online gambling, is no longer there for her even though she is going through a time when she needs support. She thinks it will be difficult to raise children with such an absent man.

Ms. T: If it gets too much, I'll leave! I have to think about myself. I'm 25 years old, I'm not 40. If at some point I think it's too much and I can't live like this, I'll leave. It makes me sad to have someone by my side who has these defects. We all have flaws, but this one is the one that prevents me from taking a step forward with Antoine and that prevents me from projecting myself with Antoine ad vitam eternam.
THERAPIST: And what would this cap be?
Ms. T: It's trust and to tell myself that all that is behind us: these switches that you can have on the game. I don't think that this bubble is useful to you for the future. I think that whatever happens, this bubble won't serve you for life! And whether it's with me or someone else!

### Making Connections Between the Feeling of Ambiguous Loss and Previous Losses

According to Stern, “you can't change without changing the functional past, that is, the past that is activated” in the interaction and that will impact the perception of what is happening in the present (Stern, 2003, p. 256). The lack of emotional availability of the dependent patient can reactivate a previous experience of loss. The death of a loved one or a painful separation is mentioned incidentally at some point in therapy. During the therapy, Ms. V never spoke about her sister's death. At the eighteenth session, she talks about the strained relationship with her parents and their opposition to her marriage with her first husband. She connotes the marriage in a negative way, referring quite unexpectedly to the death of her sister which occurred at that time. This drama could not be spoken. The therapist begins to build a hypothesis around the non-elaboration of this event.

Ms. V: It was a failed marriage because it happened just before my sister died. We didn't change anything from the wedding, we kept the ceremony, we kept the wedding date when my sister had just been buried. It was horrible! It is a great sadness somewhere! She was 25 years old.

The evocation of this event may be stressful and emotionally challenging for the therapist. Sometimes it's better to wait to approach this material. The therapist waits until the family is ready for this work because at first glance the work on the past and the transgenerational seems unrelated to the problems of TCIs. The therapist assumes that the exacerbation of an ambiguous sense of loss, related to the use of ITCs, must be linked to a previous loss. The feeling of ambiguous loss brings some continuity with the past.

### Reframing the Use of Technology as an Emotional Regulation Strategy

The therapist explores the relationship to the screens in terms of emotional regulation according to the Gross's process model (2014). Is it an escape from the real world? Procrastination? Extrication from the couple's relationship? Which emotions are regulated by the use of technology?

Ms. I explains that her virtual relationship allowed her to survive because she was suffering in her couple. She was living with difficulty the lack of closeness with her husband.
Ms. I: These stories with men are more virtual than real. It doesn't change anything to Philippe's suffering. I'm not trying to diminish the fact that I betrayed him, whether it was by texting, by fantasy. For me these stories were about survival from many different angles. I think that Philippe and I would no longer exist as a couple if I hadn't had that escapism and I think there was a real unconscious desire for Philippe to see them, I didn't erase them because I needed our couple, as it existed, to explode somehow, to disappear. and I wanted to annihilate that couple as it existed and build something else, preferably with Philippe. But I didn't want that old couple anymore! The only thing that hurts me is that Philippe is suffering! Because I know it hurts. And at the same time, I think I was ready to do anything to stop having that couple functioning. And we had long discussions with Philippe and I think it was the first time in our life together that I felt that Philippe and I were communicating, that he really heard me and that something was happening! I didn't want this couple anymore! I couldn't live in that couple anymore! So I think that was my way of shattering the whole thing! I couldn't leave him; I couldn't get our relationship to change to be harmonious. I was terribly unhappy and had been suffering for several years to the point of making myself sick with depressions, to the point of dissociating myself.

For Mr. T, online poker allows him to live with neuroticism.

Mr. T: It's almost intrinsic to my personality.
THERAPIST: What is your sensitivity?
Mr. T: (…) I am sensitive. I'm not someone who's going to cry in front of a film, but I can be quickly hurt by people's behavior toward me. It's “sensitive”… “susceptible” in fact. What people may say to me brings back bad memories from my childhood. The fact of getting into a bubble means that you won't get affected by it.
THERAPIST: The stock of bad memories, it stays in a corner?
Mr. T: Yes! Exactly!
THERAPIST: A form of anesthesia of a part you don't want to see?
Mr. T: Yes! That's why at one time I was doing quite a bit of overdoing with medication. It's also a way to anesthetize myself because when I fell asleep, I would feel a lot, etc., and it kept me awake.

### Building the Emotion Regulation Strategies Cycle With the Family

The therapist proposes a circular view of the situation with the intention of helping everyone to better understand their differences regarding emotion managing. Mr. T is seeking sensation with gambling to anesthetize negative emotions. Ms. T loses trust and is considering leaving him. Mr. T is affected in his vulnerability to loss. He gambles so that he no longer thinks she can leave. We can observe an escalation of the couple's distress. The therapist highlights the circularity of emotional regulation strategies and the impasse in which the couple or the family is stuck. At this stage, the family begins to understand the urgency of changing the emotional patterns related to the loss so that old patterns are not repeated. The therapist suggests alternative strategies. For example, in the case of the V Family, she helps Ms. V. accept that her sons do not share negative emotions.

GAUTHIER: We have very different ways of coping. I don't handle excessive expression of emotions very well. My way of dealing with emotions is rationalism. I rationalize everything. I contain myself by rationalizing. And as a result, I am never on the side of amplification.
Ms. V: As a young person, there was a major ban on expressing emotions in my family and I was very unhappy about that. I took French leave. It is as if with my boys, I was put back in the atmosphere of my childhood family. All the work I had done to free myself from this leaden screed, where it was forbidden to say anything and we had to pretend, seems useless today. I am trapped again. If I can't have intimate, intense conversations with my sons, I'm going to put myself at a distance so as not to get hurt.
THERAPIST: Emotional intensity is experienced as a danger for your sons. The further you go in the expression of your emotions, the more they flee and a gap sets up between you. It is not a question of love, it is just that you are different, with a different way of regulating emotions and you have not yet managed to accommodate with this difference.

### Opening Up Earlier Trauma Wounds

When the family is ready, the past can be put in perspective. The therapist explores transgenerational processes, with the genogram (McGoldrick et al., 2020) or the Goose Game (Colpin and Rey, 2019) or a systemic tale (Duriez, 2020). Our presupposition is that the emergence of symptoms related to pathological use of the Internet and family dynamics, sources of suffering, are the result of the weaving between transgenerational dynamics and social changes. The feeling of ambiguous loss has reactivated existing psychological wounds from the parents' lives, which intensified the emotional reactivity between the one with earlier trauma wound and the one who is locked in a technological bubble. These parents are vulnerable to loss. For the five families, we found unhealed wounds originated in childhood trauma: conflictual separation of parents and neglect (Mr. T), emotional abuse (Ms. R and Ms. V), violence within family of origin (Ms. I), abandonment (Mr. I and Ms. V), migration (Ms. S), death (Ms. V). For example, the therapist writes a systemic tale with the I family and evokes the adolescence of Mr. I when he was relegated to a maid's room. Ms. V also evokes a difficult adolescence:

Ms V: I shared a room with my sister. She laid out all her things and there was no room for two in the bedroom. I stayed 1 year $\frac{1}{2}$ sleeping on a cot in the living room. My father didn't want to do the work to create a new room. My parents rented a small maid's room for me, I stayed for a year there and then I left.

The family begins to make connections between past experiences of anger within the family of origin and current aggression. Reexperiencing trauma memories in a safe therapeutic environment is the key to changing maladaptive aspects of the memory. Such moments of high arousal appear to be imprinted in emotional memory with special potency. They result in emotional responses that can't be understood by children. The parents themselves don't understand. This leaves a person with potent emotional learned repertoires that are difficult to understand because they are stored in emotional memory in an unsymbolized manner. During therapy, anger or intense emotional reaction may need to be aroused to some degree to be reprocessed in the safety of the therapy situation.

### Building a Sense of Internal Security/or Autonomy

The therapist's interventions aim to help family members accept their differences, whether in relation to the use of technology, to their values or to their emotional regulation strategies. The therapist questions and deconstructs the “schema-of-being-with^2^” (Stern, 1985) that is activated to interpret what the other person is saying. Together they feel more emotionally secure and can address each other's emotional needs more appropriately. Speech flows more freely, with respect for the other person. The parent's empathic adjustment to the child's needs, moods and fears helps the child internalize a sense of security from the parent and this helps establish a basis for internal security in the child.

Ms. V is worried about her sons. She worries because Gauthier has no partner, she worries because Florent doesn't seem to have concrete plans for his professional future or even for the vacations. They both want to protect themselves from their mother's concern, which they perceive as a lack of understanding or trust. They react coldly to her concern. The therapist tries to deconstruct this “schema-of-being-with.”
THERAPIST: According to your mother's logic, for a vacation, it has to be planned, there has to be a program.
Ms. V: A program or a desire! I don't hear him say “Mommy I want to.” I don't hear any concrete project from you, I have a floating feeling as if you were weightless. I sent you back to that and you said, “But trust me!”
FLORENT: I had the feeling that you didn't trust what I was going to do and I find that upsetting. I just didn't feel like talking about it at the time and it wasn't really organized yet.
Ms. V questions her sons and they don't get her questions right. The interviews help Ms. V accept Gauthier's choice of life and respect Florent's path to set up his projects according to his own modalities.

### Implementation of New Behaviors

In this more secure environment, family members can experiment with new ways of communicating. Together they build new narratives of their experiences. Ms. V. and her sons build a common narrative about the place of video games in the family. They agree that the withdrawal into video games is a consequence of the conflictual atmosphere that existed at the time.

GAUTHIER: Video games were very far from what we could call an addiction, it was more of a passion, this was confirmed afterwards. Florent, like me, we continue to do quite a lot, it's a substantial part of our leisure time and my conception of addiction, it doesn't have the characteristics of it, neither before nor now. When Mom started things from that point of view, it seemed absurd to me!
THERAPIST: Madam, do you make a link between playing video games and communication problems?
Ms. V: Yes, I do.
GAUTHIER: A link what? A causal link? A link of consequences? A correlation link?
Ms. V: I don't have the same vision. Compared to some young people, you're not in a total addiction, spending nights and days playing, that's for sure, you manage to have your social life, to see friends, to work, etc. But for all that, at one time, it took up a lot of space on a time when we could have spent it together and we couldn't. I'm just saying that it was also a consequence of a mode of communication, of living together, a way of communicating, or also of conflicts, perhaps of things that we hadn't said to each other or that weren't resolved, that made you fall back on friends and video games essentially. There were no exchanges, no sharing time more than that.
GAUTHIER: I agree that it was a consequence of an atmosphere, a way of life, rather than a cause.
Previously repressed emotions are expressed. Gauthier can express his sadness at the old family functioning, which he describes as “deleterious.”
GAUTHIER: It was really a way of life that had settled down and an atmosphere that was perpetuated, it was a vicious circle that continued to turn and that was harmful for everyone. The best way to break it was to leave.

Together they build new family rituals. In the context of quarantine, they are in different geographical locations. Consultations are held by videoconference every month. At the same time, Gauthier initiates videoconference appointments with his mother and brother every week. Technology now fosters the family cohesiveness.

### Toward the Acceptation of Emotions and the Affirmation of a Stronger Self

The intent of the therapist is to help each person use emotion regulation in a flexible manner and in a way that is consistent with their goals and is considerate of the needs of others. We expect that family members gain emotional intelligence, learn to review the context before deciding whether and how they should regulate their emotion and take into account and maximize intra-individual and inter-individual long-term welfare. For well advanced therapies, some may have reflected on what was passed down to them by their parents or by previous generations. They know that they can refuse this legacy. They have a better understanding of how “schemas-of-being-with-the-other” were created during their childhood and are ready to build new ones. This awareness increases the agency in one's own life and goes in the direction of the emergence of a stronger self. In the last session, Ms. V summarized her change as follows:

Ms V: Now I can take a step back from the situation, to look at what is happening with fresh pair of eyes, a little bit detached from the scene. Then, I can get out of something epidermal, emotional, to really see what's going on for them. The feelings I had with regard to the wounds, allowed me to take a step back from them more: it's not very easy but it's less painful. From the moment, I can better understand what is happening to the other person, see them as part of a different world, in different relationships, and this makes it a lot it easier. Understanding the other is important.
Ms. I asserts herself through writing. Ms. R practices hiking. Mr. T understands his gambling behavior as an emotion regulation strategy and uses alternative strategies, supported by his wife. Ms. S accepts that her husband will not change, does meditation to accept her sadness toward her couple and her worries about her children: “I do meditation, I take distance, I let go in the sense that I know that I cannot change him.” She has the therapist listen to a podcast on accepting emotions.

## Discussion

The current study reports on a change process research focused on therapist interventions with five wired families. We can say that these families are healthy, there are no severe symptoms within these families. The internet use is problematic but life didn't stop. Teenagers go to high school. Young adults and parents go to work. These five families are contemporary families who are challenged by the effects of new technologies on their organization. More severe situations, for example with a teenager locked in his room night and day, would probably require a different approach, more focused on the subject and his psychic vulnerability. The therapist's interventions highlight family dynamics which are consistent with previous research which has shown that PIU and interpersonal relationship problems have a strong correlation. The family approach then seems indicated, which is in line with the recommendations of Young Park et al. (2014) for adolescents.

While 12 phases are presented for these interventions, there is fluidity in the therapeutic process that honors the family's priorities and dynamics as the point of entry. The interventions follow the family's focus, not necessarily in a prescribed linear progression but in a systemic fashion. The family therapist looks into family interactions that maintain problematic behaviors: transactional patterns (Minuchin, 1974 and First-Order Cybernetics), “official program” and map of the world (Elkaim, 1986 and Second-Order Cybernetics) and emotion regulation patterns (Duriez, 2017 and Third-Order Cybernectics). Behavior, cognition and affect work together to create specific interactions which contribute to intergenerational transmission. This consideration of emotion is not new (Bowen, 1978; Johnson, 2004; Greenberg, 2015) but with research in neuroscience and epigenetics, we now have a better knowledge of the complexity of emotional regulation strategies, which is essential for a good perceptivity of circularity in interpersonal relationships. Our work has therefore focused on emotional regulation strategies, whether intentional or automatic, to better identify mechanisms of homeostasis. Bowen (1978) taught us that the lower the level of differentiation is, the more difficult it will be to regulate negative emotions. In the families studied, we observe a low level of differentiation. In fact, we can consider escapism into a technological bubble as a triangulation process that regulates stress, anxiety or tension. Bowen found that tension in a two-person relationship is relieved by focusing on a third person. Today the third party can be a computer, a tablet or a smartphone. Our clinical practice is influenced by the Bowen Model to help the patient identify their relationship to the technological object as a triangulation process, avoiding communication about a problem.

Corroborating previous findings (Lee, 2014), underfunctioning, uninvolvement, and the irresponsibility of the father in areas of parenting and household chores appeared in four of the five families. For the fifth one, the parents are separated. The over-functioning partner was commonly female. We cannot link this underfunctioning to PIU. In the case of the I family, it is the woman who has a PIU but this use does not prevent her from carrying out her parenting responsibilities, at times she could even appear as too invasive according to her children. She builds an intimate emotional cyber relationship with another man to escape from a feeling of abandonment, to forget the lack of connection with her husband and their problem relating to intimacy. Men escape from family responsibilities and rely on women. Women have to cope with a higher mental load and feelings of loneliness. We find the pattern: “The over-functioning spouse was often other-focused in contrast to the excessive self-focus of the underfunctioning partner” (Lee, 2014, p. 381). Within this framework where the woman gives and the man takes, Contextual Family Therapy can help to consider the balance of fairness in family relations. But it's really difficult with the excessive self-focus men. As Lee we were seeing rebuffs and the absence of acknowledgment by the men (M. I. and M. S.). “Shame and a feeling of unworthiness also curtailed self-disclosures” (Lee, 2014, p. 379). This was not the case for the T couple, perhaps because of their youth. When Ms. S tries to express herself, her husband, unable to connect with her emotion, empathy, ends up leaving the session. It seems biologically impossible for him to take his wife's needs into account. An exploration of the literature on dynamic self- and other-focused emotional intelligence (Pekaar et al., 2020) may allow us to refine interventions.

According to the literature Mesch (2006), our findings show that the main problem challenged by the families is the time spent on the Internet to the detriment of family time. “Family time is a major component contributing to family cohesion. It facilitates the development of a collective identity and shared experiences” (Mesch, 2006, p. 125). Weak family cohesion, lack of communication and loss of rituals reduce the perception of family belonging and increases individuality. This creates suffering (Crosnoe and Elder, 2004; Cavanagh, 2008; King et al., 2016). This suffering may be amplified by the presence of old wounds. Can we then consider these relational patterns, with a reorganization of family time, as a new challenge for contemporary families? Is this a specific challenge for wired families? We can assume that if there was no such possibility to escape from negative emotions with technology, there would be an escape by other means (working, playing music, reading, practicing sports, gardening, tinkering, housework, etc.). Already 50 years ago teenagers locked themselves in their room to listen to music or read to extricate from the relationship with their parents. Men went out to see friends when they didn't want to spend the evening with their wife. What is new is that technology is within reach, in the household, and offers very attractive and addictive activities.

Therapeutic accompaniment must therefore make it possible to find the meaning of this problematic use, rather than eradicating it without taking into account the function of this use in terms of emotional regulation. This emotional regulation strategy which can be procrastination for Mr. S who watches series instead of working, an escape for Baptiste, Simon and Lucas R, for Logan S, for Gauthier and Florent V, for Ms. I and her son, Axel, and an anesthesia for Mr. T. As Bowen (1978), we assume that the past influences the present, as past relational patterns continue to influence the present family system. If the family has become rigid in its organization where avoidance strategies dominate, can it be a way of dealing with grief or separation from previous generations? This refocusing on transgenerational processes helps the system to become more flexible, to stop blaming and to assume its own role in this rigid functioning. As therapist and family members see how patterns repeat over generations, it is possible to identify the automatic reactions of family members toward each other. The ability to act on the basis of more awareness of relationship process can lead to some reduction in problematic internet use.

Our approach is similar to that of D'Amore who also worked on the impact of losses on contemporary families: “The family suffering from breakups could freeze in on itself and organize itself as a grieving relational system where the loss would constitute the organizing core of its identity and experiences” (D'Amore, 2010, p. 17). We suppose that these families where dependence on technological objects is such that it makes some people experience a sense of ambiguous loss are families that are self-organized around the loss: some behaving in such a way as to make others experience this feeling, while others experience this sense of ambiguous loss. In this way, family identity is maintained generation after generation.

The study has several limitations. Great caution should be taken when generalizing the results. Further research with larger and more homogeneous samples (e.g., recruitment of families with the same problem) and studies that test the ecological validity of interventions listed in this study are suggested. This research does not investigate the effectiveness of the model presented. It is only an exploratory research, conducted in natural clinical conditions. This is why the population is heterogeneous as it exists in the active file of the center. The duration of care is not the same. The T family stopped after two sessions because they found a positive dynamic. Ms. T expressed herself and felt heard. The birth of the baby brought them into a new dynamic. Confidence has returned. The R family did not come back after Simon left for another region for his studies. The individual follow-up of Baptiste in a partner center continued. The three other therapies are longer.

It may be interesting to conduct separate research with larger populations and separate therapists on each of the problems presented: video game addiction for adolescents, work addiction for adults, cyber infidelity and poker addiction. This would allow us to assess whether it is relevant to specify family therapy process for each problematic internet use and to build an innovative therapeutic support for each of these clinical situations.

Third, this study only focused on the therapists' interventions without taking the family's behaviors into consideration. Data on family dynamics were collected in a therapeutic context. However, the therapist's objective is not the same as the researcher's objective. An evaluation of family dynamics with standardized tools, such as the evaluation of cohesion with the FACES IV, would have been more rigorous.

The therapist-researcher works with her own subjectivity. The therapist's self is an important factor in the therapeutic process. We have not explored this subjectivity here. The choice to work on emotional regulation strategies and the relationship that the therapist herself has with connected objects deserves to be explored. We suggest further research concentrating on the interaction between therapists and clients in this context. We examined before how the therapist regulates their own emotions regarding the problem brought by the family within the therapeutic encounter. In this way they offer the family an alternative model of emotional regulation that introduces a difference within the system. The therapist's emotional regulation strategies are a core component of the therapeutic process (Duriez, 2018b).

Rather than categorizing the therapist's interventions with grounded theory, it might have been interesting to build five distinct intensive case studies (Duriez, 2009) in order to tackle clinical case's complexity and to identify therapeutic process in all its singularity for each family. We should track changes in family members' constructions over the course of the entire treatment for each family, with measurement of the client's problems, for example with the Generalized Problematic Internet Use Scale 2 (GPIUS2; Caplan, 2010), and with The Difficulties in Emotion Regulation Scale (DERS; Gratz and Roemer, 2004) at different times. Post-treatment interviews with members of the families could provide another perspective.

## Conclusion

These findings led to propositions for an innovative therapeutic support in family therapy, the *Emotion Regulation Focused Family Therapy* with wired families. Therapeutic work has two directions: (1) the problematic internet use as a maladaptive self-regulatory strategy and its consequences for the addicted person as well as for their entourage according to the contextual approach and (2) the elaboration of a previous loss for the suffering person in order to have a stronger self, regain autonomy and get out of co-dependency. We hope that this study will enrich the practice of family therapists with wired families.

## Data Availability Statement

The data analyzed in this study is subject to the following licenses/restrictions: The datasets analyzed during the current study are not publicly available due to confidentiality but are available from the corresponding author on reasonable request. Requests to access these datasets should be directed to nathalie.duriez@iedparis8.net.

## Ethics Statement

The research presented in this manuscript was conducted using the records of five families whose therapy has been completed. It's a retrospective non-interventional research. Written informed consent for the use of these data for research, in particular video recordings, was collected from all the members of family. They were informed that the data may be used for practice analysis and change process research. They were informed they were free to request that the recordings be erased should they change their mind. They were assured they would remain anonymous and fictitious names are used in this text as a safeguard. Ethical review and approval was not required for the study on human participants in accordance with the local legislation and institutional requirements.

## Author Contributions

ND was the principal investigator who formulated the research question, developed the study protocol, conducted data analyses, and wrote the manuscript.

## Conflict of Interest

The author declares that the research was conducted in the absence of any commercial or financial relationships that could be construed as a potential conflict of interest.

## Footnotes

^1^Family cohesion is defined as the “emotional bonding that family members have toward one another” (Olson et al., 1983, p. 60).

^2^The schema of a-way-of-being-with is conceptualized by Stern from the assumed subjective perspective of the infant in interaction with the caregiver.
